# *Scorzonera undulata*: Traditional Applications, Phytochemical Analysis, and Biological and Pharmacological Attributes

**DOI:** 10.3390/plants14111606

**Published:** 2025-05-24

**Authors:** Mohammed Ajebli, Ayoub Amssayef, Maryame Sabiri, Fatima Zahrae Radi, Eimad Dine Tariq Bouhlali, Mohamed Eddouks

**Affiliations:** 1 Faculty of Sciences and Tecniques Errachidia, Moulay Ismail University, Meknes 11201, Morocco; mohammed.ajebli@gmail.com (M.A.); mohamed.eddouks@laposte.net (M.E.); 2 Laboratory of Biotechnology, Conservation and Valorization of Bioressources (BCVB), Research Unit: Api-Phytotherapy, Physiology, Environnement and Health, Department of Biology, Faculty of Sciences Dhar El Mahraz, Sidi Mohammed Ben Abdellah University, Fez 30003, Morocco; a.amssayef@ueuromed.org; 3 Laboratory of Molecular Chemistry and Natural Substances, Faculty of Sciences, Moulay Ismail University, Meknes 11201, Morocco; sabirimaryame@gmail.com; 4 Higher Institute of Nursing Professions and Technics of Health (ISPITS), Fez 30050, Morocco; fati_radi2007@hotmail.com; 5 Oasis Systems Research Unit, Regional Center of Agricultural Research of Errachidia, National Institute of Agricultural Research, Avenue Ennasr, BP 415 Rabat Principale, Rabat 10090, Morocco

**Keywords:** *Scorzonera undulata*, traditional medicine, biological activities, pharmacological proprieties, toxicity

## Abstract

*Scorzonera undulata* (*S. undulata*) is a medicinal plant that is traditionally used to treat various health conditions, including diabetes, constipation, diarrhea, and other digestive issues. However, comprehensive analysis of its traditional uses, phytochemistry, and pharmacological applications is still lacking. This review aims to systematically consolidate available information on the ethnopharmacological relevance, chemical profiles, and pharmacological activities of *S. undulata*. A comprehensive literature review of *S. undulata* was conducted across multiple scientific databases. Based on predefined inclusion criteria (full-text English publications providing relevant data on *S. undulata*) and exclusion criteria (abstracts only, studies on other species), 29 relevant studies were selected. This review systematically integrated traditional ethnobotanical knowledge with modern scientific insights, analyzing phytochemical compositions, biological activities, and pharmacological potential through a methodology designed to ensure unbiased selection from diverse sources. Traditional uses of *S. undulata* include treatments for diabetes, gastrointestinal disorders, snake bites, dehydration, and burns. Phytochemical studies revealed a wealth of polyphenols, flavonoids, tannins, glycosides, terpenoids, and sesquiterpenoids. In vitro and in vivo assays showed antibacterial, antifungal, anti-inflammatory, antidiabetic, antihypertensive, cytotoxic, and antioxidant properties. There are insufficient toxicity studies to assess the safety of this species. However, pharmacological research on this species remains limited. This review is the first to synthesize the traditional uses, phytochemistry, and biological activities of *S. undulata*, highlighting its pharmacological potential. However, further comprehensive research, including clinical trials, toxicological evaluations, and mechanistic studies, is necessary to fully identify active compounds and confirm their therapeutic applications, thus warranting additional investigation into this medicinal herb’s complete benefits.

## 1. Introduction

The genus *Scorzonera* L. belongs to the tribe *Lactuceae*, within the family Asteraceae. First documented by C. Linnaeus in “*Species Plantarum*” (1753) as one of the most primitive taxa, *Scorzonera* comprises approximately 175 species of mostly perennial herbaceous plants [1]. These plants, originally from the ancient Mediterranean, are now widespread in temperate and arid regions across Central Europe, Central Asia, and North Africa [2]. *Scorzonera undulata* (*S. undulata*), also known as wavy-leaved salsify, is native to North Africa, Southern Europe, and Western Asia. This perennial herb is characterized by erect, branched stems (1–3 feet high); alternate, lanceolate leaves with wavy edges; and long, black, arched tubers [3,4,5].

*S. undulata* has a rich history of traditional medicinal use, particularly in the Mediterranean region. Ethnobotanically, it has been valued for diverse therapeutic properties, with different plant parts used to treat various ailments [6]. The roots or tubers have been employed as a diuretic, antihypertensive, and to manage digestive issues like stomach pain and constipation. The leaves have been used for their anti-inflammatory and wound-healing properties, while the flowers have been used for their purported antispasmodic and sedative effects. This long-standing traditional use highlights the plant’s versatility and therapeutic potential [7,8].

Research interest in *S. undulata* stems from its diverse pharmacological activities. Extracts have exhibited antidiabetic, anticancer, and hepatoprotective properties in experiments, potentially through mechanisms such as modulating cell signaling and gene expression [9]. The plant’s antioxidant and anti-inflammatory activities may be beneficial in managing conditions involving oxidative stress and inflammation, and its antimicrobial abilities suggest potential as an alternative to conventional antimicrobials [10,11].

Validating traditional knowledge in modern pharmacology, particularly for *S. undulata*, presents several challenges. Traditional applications often stem from generations of empirical observation, lacking the standardized protocols and controlled clinical trials typical of modern scientific research. The inherent variability in plant composition, influenced by environmental factors, agricultural practices, and extraction methods, further complicates efforts to reproduce and standardize pharmacological studies. Moreover, reconciling ethnobotanical information with scientific evidence demands interdisciplinary collaboration, integrating phytochemical analysis, bioactivity assays, and pharmacokinetics studies. Beyond these scientific hurdles, ethical considerations are paramount. These include protecting the intellectual property rights of traditional knowledge holders and ensuring equitable acknowledgment and benefit-sharing in the development of new therapies. Effectively addressing these challenges is crucial for both validating traditional knowledge and fostering its responsible integration into modern medicine.

This review seeks to provide a thorough overview of the current understanding of this plant’s traditional medicinal applications, as well as its phytochemical composition and the pharmacological activities that have been investigated. By consolidating the available data from diverse studies, this review aims to shed light on the untapped potential of *S. undulata* and its possible therapeutic applications. The analysis of ethnopharmacological information, coupled with the evaluation of scientific research, can contribute to the validation of traditional knowledge and guide future investigations into the development of novel phytotherapeutic approaches utilizing this understudied medicinal plant.

## 2. Methodology

Four researchers conducted a comprehensive literature review (14 April 2023 to 22 January 2025) across databases including Google Scholar, Elsevier, Medline, Web of Science, Scopus, and PubMed. We focused on ethnobotany, phytochemistry, taxonomy, pharmacology, toxicity, and clinical studies of *S. undulata*, using specific search terms. Out of 81 references, 29 relevant articles were selected, and chemical structures were visualized using ChemBioDraw Ultra (V16.0).

### 2.1. Inclusion and Exclusion Criteria

The inclusion criteria encompassed full-text papers published in English that provide relevant ethnobotanical, phytochemical, biological, and pharmacological data on *S. undulata*. The exclusion criteria included studies available only as abstracts, research focusing on species other than *undulata*, and studies addressing topics beyond ethnobotanical, phytochemical, biological, or pharmacological aspects.

### 2.2. Rationale for Study Choices and Methods Used to Assess the Quality or Risk of Bias

The rationale for the selection of the studies herein lay in the necessity of integrating traditional wisdom with modern scientific information. The present review seeks to reconfirm the therapeutic promise of *S. undulata* and foster its potential uses in contemporary medicine through investigation of its traditional uses, screening for bioactive compounds using phytochemical analysis, and evaluation of its biological and pharmacological activities. A systematic method was used to reduce bias while selecting the literature. A wide variety of sources, such as peer-reviewed papers, credible books, and authentic databases, were searched to ensure an extensive exploration of the subject of interest. Care was taken to include studies from various geographic regions to give a balanced view of indigenous as well as foreign views regarding this species. To avoid publication bias, both positive and negative findings that represented a broad variety of methodologies, including in vitro, in vivo, and clinical studies, were included in the review. It is crucial to mention that two researchers independently screened the literature to choose the relevant studies, yielding a comprehensive and unbiased selection. The duplicate entries were subsequently excluded carefully to prevent overrepresentation of any study and maintain the integrity of the analysis. Clear study inclusion and exclusion criteria were well documented to improve clarity and increase the credibility of the reported results.

## 3. Results and Discussion

### 3.1. Taxonomy

*S. undulata* Vahl belongs to the *Asteraceae* family, one of the most extensive groups of flowering plants worldwide, comprising over 1600 genera and 25,000 species [12]. The genus *Scorzonera* encompasses nearly 200 species, naturally occurring in Europe, Eurasia, and Africa, particularly in arid regions [13]. *S. undulata* is also known by another name: *Pseudopodospermum undulatum*. However, this species includes two subspecies: *S. undulata* subsp. *deliciosa* (Guss.) Maire and *S. undulata* subsp. *undulata* [14].

The *Practical Flora of Morocco* recognizes two subspecies of this plant: Subsp. deliciosa is described as smooth (hairless) and fragrant. Subsp. *undulata* is described as hairy and lacking any fragrance. However, the botanist René Charles Maire observed an additional distinction in his 1931 publication [15].

### 3.2. Geographic Distribution

*S. undulata* flourishes in hilly regions, particularly in sandy clay alluvial soils and pastures. This plant is commonly found in North African countries such as Morocco, Algeria, Tunisia, Libya, and Egypt, as well as in several European countries, including France, Spain, and Italy. Additionally, *S. undulata* is prevalent in certain Asian countries, namely, the Syrian Arab Republic, Lebanon, and Turkey [16].

In Morocco, *S. undulata*, a perennial plant, has evolved remarkable adaptations that allow it to thrive even in the face of challenging environmental conditions. This species takes advantage of the slightest rainfall events to soak its tuber with water, enabling it to bloom and flower almost every year [17]. Unlike many annual plants that require consistent, adequate rainfall to complete their life cycle, *S. undulata*’s perennial nature and ability to store water in its tuber give it a distinct advantage in arid or semi-arid regions with erratic or sparse precipitation patterns. In spring, this plant transforms even the most barren and empty areas with its blooms, often the only flower to be seen [18].

### 3.3. Botanical Description

*S. undulata* is a perennial plant characterized by a thick, blackish stem, which can be consumed during the springtime. The leaves grow in clusters, with a narrow, elongated shape that is wavy at the edges, featuring a glaucous color and covered with short, woolly hairs. The stems are typically simple and short (10–30 cm), with bare peduncles that terminate in a capitulum. The capitulum is large, measuring 3–5 cm in length and diameter when open, surrounded by large, uneven membranous bracts with long, purple-rust ligules at the edges. The flowers are ornamental, opening in the sunlight and closing in the shade. The lower portions of the slightly ribbed achenes are somewhat swollen [19].

### 3.4. Ethnomedicinal Uses

The use of medicinal plants in the pharmacopeia of the Mediterranean region has a long history, dating back to ancient times. Countries like Morocco, Tunisia, Algeria, Greece, Italy, Spain, and Turkey have rich traditions of utilizing herbs and plants for their healing properties [20]. These plants are often incorporated into traditional medicines, herbal remedies, and even culinary practices. The warm climate of the Mediterranean region supports a diverse array of plant species with medicinal qualities, such as lavender, rosemary, oregano, and olive trees. The knowledge of these traditional remedies has been passed down through generations and continues to play a significant role in the healthcare practices of the region [21]. Table 1 showcases the various documented uses of *S. undulata* across its native regions.

*Scorzonera* species are recognized by their potential uses in traditional medicine, particularly in the Mediterranean region. In Turkish folk medicine, *Scorzonera* species are utilized as medicinal plants for ailments such as arteriosclerosis, kidney diseases, hypertension, diabetes mellitus, and rheumatism [29]. Moreover, they are utilized in European traditional medicine to combat pulmonary diseases and colds, heal wounds, address gastrointestinal issues, and promote stomach health. They are also known for their diuretic, galactogogue, antipyretic, and appetizing properties [30,31].

**Figure 1:**
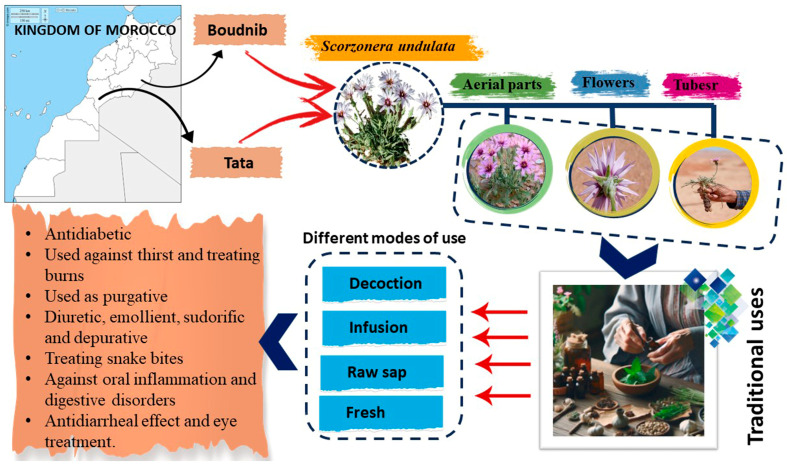
The geographical distribution of *S. undulata* in Morocco and its traditional uses in various regions where it naturally thrives.

The ethnobotanical applications of *S. undulata* have been well documented, highlighting its diversity of medicinal properties (Figure 1). Traditions indicate that the aerial portions are commonly used as a livestock diuretic in rural communities, although seasonal cautions apply due to potential toxicity risks if consumed during the summer months [28].

Recent studies provide further insights into potential health benefits. Aqueous extracts of aerial parts exhibited significant antihyperglycemic effects in experimental models, suggesting safety based on preliminary animal studies [32]. Separately, tuber extracts demonstrated notable hypolipidemic, antioxidant, and cardioprotective activities—particularly in streptozotocin-induced diabetic rodents [33]. Beyond pharmacological uses, *S. undulata*’s rich phytochemical composition is attracting taxonomic investigation for insights into chemotaxonomy patterns across the *Scorzonera* genus. A holistic perspective contextualizes its ethnobotanical usages and underscores its broader therapeutic potential.

By synthesizing traditional knowledge with modern scientific research, *S. undulata* is emerging as a multi-faceted botanical resource. Preliminary findings suggest opportunities for both traditional medicinal applications and contemporary drug discovery approaches leveraging its diverse biological activities. Further validation is still needed, but the initial results highlight *S. undulata*’s promising prospects, deserving of continued exploration.

Various parts of *S. undulata* are consumed as food, either raw or cooked [1]. Additionally, *S. undulata* has traditionally been utilized in medicine to address various ailments (Table 1). In Moroccan traditional medicine, raw flowers are ingested to manage diabetes mellitus and snake bites [16]. In Tunisia, the ash from burned *S. undulata* tubers is effectively applied to soothe and treat burns [34]. Furthermore, Idoudi et al. (2023) reported the use of a decoction of this species as a purgative agent in Tunisia [18]. In Algerian folk medicine, roots and infused leaves are employed as diuretics, emollients, sudorifics, and depuratives [25,35]. Brahim and their co-workers documented that the subspecies *S. undulata* ssp. *deliciosa* is utilized in the treatment of snake bites. Moreover, the leaves of this subspecies are used as a laxative to manage constipation within the Libyan population.

*S. undulata* is a popular edible plant that is commonly consumed in regions where it is grown. The leaves of this plant are commonly consumed by steaming them and mixing them with vegetable butter or olive oil [36].

### 3.5. Phytochemical Properties of S. undulata

Enhancing the quality of research on medicinal plants relies heavily on conducting in-depth phytochemical investigations. It is imperative to thoroughly characterize, identify, and isolate the phytochemical compounds present in these plants before commencing any biological or pharmacological studies. Table 2 below provides a comprehensive overview of the phytochemical analysis conducted on *S. undulata*, capturing all relevant data known to us at this time.

Phytochemical analysis of medicinal plants plays a vital role in determining their biological, pharmacological, and industrial significance. By identifying the active chemical constituents in these plants, researchers can better understand their medical applications and explore potential uses in various industries.

As shown in Table 2, numerous studies of *S. undulata*’s phytochemistry have been conducted by scientists from different academic and research institutions around the world. Various extraction and analytical techniques have been employed to profile the secondary metabolites present in this species. The results have revealed a wide variety of bioactive compounds, including terpenes, phenolics, flavonoids, and other phytoconstituents. For example, essential oil analysis by gas chromatography–mass spectrometry has identified monoterpenes and sesquiterpenes as major volatile components. Methanolic extracts have been found to contain caffeic acid derivatives, polyacetylenes, and lignans after chromatographic isolation and structural elucidation using NMR spectroscopy and mass spectrometry. Other work has characterized unique flavonol glycosides that are only present in *S. undulata*. This extensive phytochemical profiling has provided valuable insights into *S. undulata*’s traditional ethnomedicinal uses and physiological effects. The identified bioactive compounds may be further studied for their pharmacological properties and potential drug-like activities. Overall, the accumulated data from diverse phytochemical investigations, as summarized in the table, have increased the scientific understanding of *S. undulata*’s chemical makeup and aided efforts to uncover new applications for this important medicinal plant species.

*S. undulata*, a medicinal plant that is native to various regions, has been studied for its phytochemical composition. The aerial parts and roots of this plant contain a diverse array of bioactive compounds, which have been extracted using different methods and solvents. Maceration with methanol was the most commonly used extraction technique, yielding 0.78% and 0.86% extracts from the aerial parts and roots, respectively. These methanolic extracts were found to be rich in polyphenols, flavonoids, and tannins. HPLC analysis of the aerial parts revealed the presence of apigenin-7-glucoside, gallic acid, luteolin-7-glucoside, and p-coumaric acid, while the roots contained luteolin and chlorogenic acid. Further analysis of the roots using chloroform as the solvent identified additional compounds, such as β-amyrin acetate, methyl oleanate, stigmasterol, β-sitosterol, galangustin, coumarin-O-β-glycoside, and acteoside. The aerial parts were also subjected to steam distillation, followed by chloroformic extraction. This process yielded a complex mixture of oils, hydrocarbons, terpenoids, aromatic compounds, fatty acids, and fatty acid esters. GC/MS analysis identified several key compounds, including octadecane, farnesyl acetate, benzyl salicylate, methyl hexadecanoate, heneicosane, methyl octadecanoate, and methyl linolenate. Maceration with petroleum ether and ethyl acetate was used to extract triterpenoids and flavonoids from the aerial parts. Spectroscopic methods revealed the presence of lupeol, 24-methylenecycloartanol, 3-O-(6-O-acetyl-β-D-glucopyranosyl) β-sitosterol, daucosterol, and apigenin. Meanwhile, the roots were also extracted using chloroform and methanol, yielding polyphenols and flavonoids. Column chromatography and MPLC techniques identified acteoside and galangustin as the main compounds.

#### 3.5.1. Nonvolatile Compounds

These findings highlight the rich phytochemical diversity of *S. undulata*, with the plant parts containing a wide range of bioactive compounds, including polyphenols, flavonoids, terpenoids, and fatty acids. This comprehensive phytochemical profile provides a solid foundation for further research into the therapeutic potential of this medicinal plant.

Among the principal compounds discovered in this species, we find apigenin-7-glucoside, gallic acid, luteolin-7-glucoside, and p-coumaric acid. Additionally, chlorogenic acid, galangustin, coumarin-O-β-glycoside, and acteoside contribute to the plant’s chemical profile.

Research has been conducted on the methanolic extracts from both the above-ground and root components of *S. undulata*, a plant from the *Asteraceae* family, commonly referred to as Elgiz in Libya. The findings reveal the existence of certain secondary metabolites, including coumarines, flavonoids, tannins, and various kinds of glycosides, such as phenolic, anthraquinone, and cardiac glycosides [27]. Similarly, Ajebli et al. reported that both aerial parts of this species contain polyphenols, flavonoids, tannins, glycosides, sterols, saponins, and alkaloids [33], whereas the aqueous extract of the tubers contains polyphenols, flavonoids, tannins, and glycosides [32].

*S. undulata* is renowned for its abundant array of phenolic compounds. These specialized metabolites comprise a significant portion of *S. undulata*’s phytochemical profile and contribute greatly to its renowned medicinal and nutritional properties. Its principal phenolic constituents include flavonoids, phenolic acids, and tannins. Extensive research has demonstrated that the flavonoids, phenolic acids, and tannins in *S. undulata* exhibit potent antioxidant, anti-inflammatory, and antimicrobial activities in vitro and in vivo. The powerful antioxidant capabilities of *S. undulata*’s phenolics play a crucial role in neutralizing damaging free radicals and reactive oxygen species that contribute to oxidative stress. By mitigating oxidative stress at the cellular level, the phenolic compounds help protect against acute and chronic diseases associated with oxidative damage, such as cancer and cardiovascular disorders. Experimental evidence also indicates that certain flavonoids and phenolic acids from *S. undulata* exert anti-inflammatory effects by modulating inflammatory mediators’ release and inhibiting pathways involved in the inflammatory response [41]. Additional preclinical investigations have revealed promising antimicrobial properties against an array of pathogenic bacteria and fungi [42].

**Figure 2:**
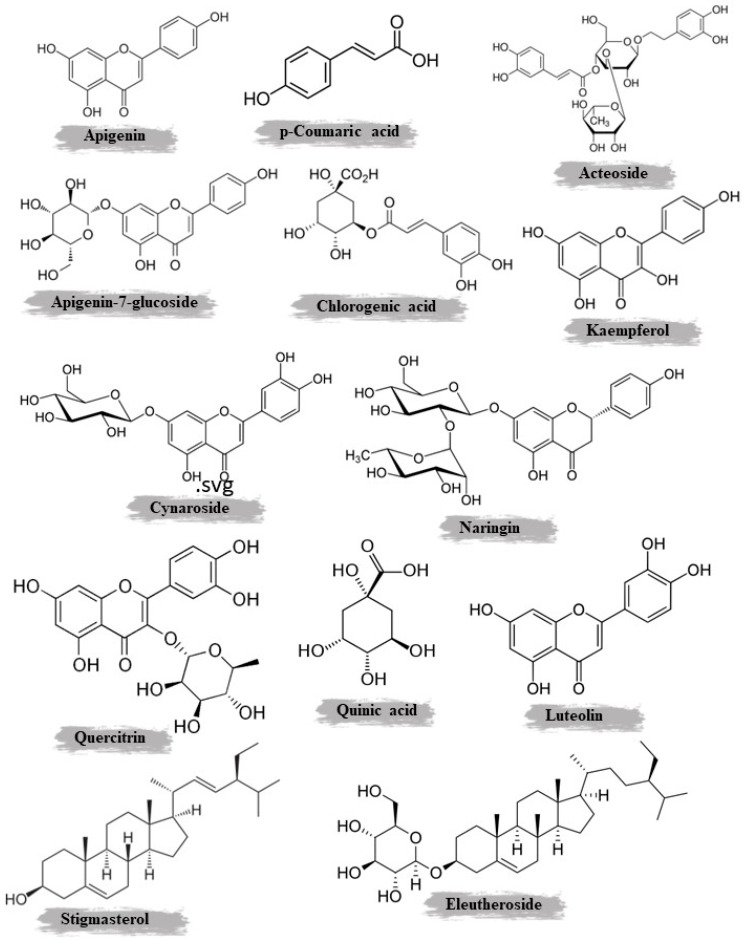
Chemical structure of phenolic molecules identified and isolated from *S. undulata*.

Erden et al. examined the phytochemical composition, mineral contents, and antioxidant properties of three *Scorzonera* species. Analysis of the aerial plant parts revealed the presence of various vitamins, including vitamin D, vitamin K, and alpha-tocopherol. A range of flavonoids were also detected, such as rutin, myricetin, morin, quercetin, and kaempferol. Phytosterols like ergosterol, stigma sterol, and beta-sitosterol were present. The sugars arabinose, fructose, glucose, sucrose, and maltose were found as well. Mineral analysis showed that the species contained varying amounts of calcium, sodium, potassium, iron, manganese, zinc, and magnesium. Copper, cobalt, and nickel were not detected. When evaluating the antioxidant effects, the extracts demonstrated antioxidant activity that increased with higher dosages. This research provides insights into the nutritional composition and potential health benefits of these *Scorzonera* species based on their phytochemical, mineral, and antioxidant profiles. The results indicate that these plants may serve as sources of vitamins, minerals, and antioxidants [9]. Figure 2 illustrates the chemical structure of important phenolic compounds identified and isolated from *S. undulata*.

Acıkara et al. (2025) performed phytochemical studies on *Scorzonera parviflora* Jacq. roots. The findings revealed three phenolic acid derivatives—namely, parvifloric acid, B, and C—and one new sesquiterpene lactone (parviflorin), together with seven known compounds that were isolated and identified as scopoline, scopoletin, caffeic acid, protocatechuic acid, 4,5-O-dicaffeoylquinic acid, 3,5-O-dicaffeoylquinic acid, and 3,5-O-dicaffeoylquinic acid methyl ester [43]. Furthermore, LC-PDA-MS and GC-MS analyses of *Scorzonera hispanica* seeds revealed the structures of several major compounds, including D-chiro-inositol, β-sitosterol, methyl 3,4-dimethoxycinnamate, caffeic acid, cis-3,5-dicaffeoylquinic acid, linoleic acid, palmitic acid, and oleic acid [44].

#### 3.5.2. Volatile Compounds and Oils

Table 2 reveals that different parts of *S. undulata* (leaves, flowers, and tubers) contain a natural store of volatile compounds. Among these compounds, we can cite the following:

β-Amyrin, a pentacyclic triterpenol, is commonly found in nature. It has been extracted from a variety of plant sources, including epicuticular wax. Within plant biosynthesis, β-amyrin serves as the precursor to oleanolic acid [45]. A research study showed that β-amyrin has enduring pain-relieving and anti-inflammatory effects in two models of chronic pain by activating cannabinoid receptors CB1 and CB2 and blocking the production of cytokines, as well as the expression of NF-κB, CREB, and cyclooxygenase 2 [46].

Stigmasterol is a phytosterol with unsaturated bonds that can be found in the fats or oils of various plants, including soybeans, Calabar beans, and rapeseed [47]. Being one of the prominent phytosterols, stigmasterol is among the sterol compounds found in the diet that have the potential to lower the risk of cardiovascular diseases [47]. Stigmasterol has been demonstrated to have anti-angiogenic and anticancer properties by reducing TNF-alpha and VEGFR-2 expression [48].

Acteoside, found in various plant species, is a significant bioactive natural compound containing caffeic acid, glucose, rhamnose, and phenylethyl alcohol. It exhibits a range of bioactivities, including anti-inflammatory, antioxidant, neuroprotective, and anti-tumor properties [49].

Heneicosane serves as a pheromone for queen or king termites of the species *Reticulitermes flavipes*. It also has the ability to lure mosquitoes of the *Aedes* genus and is effective in mosquito bait [50].

**Figure 3:**
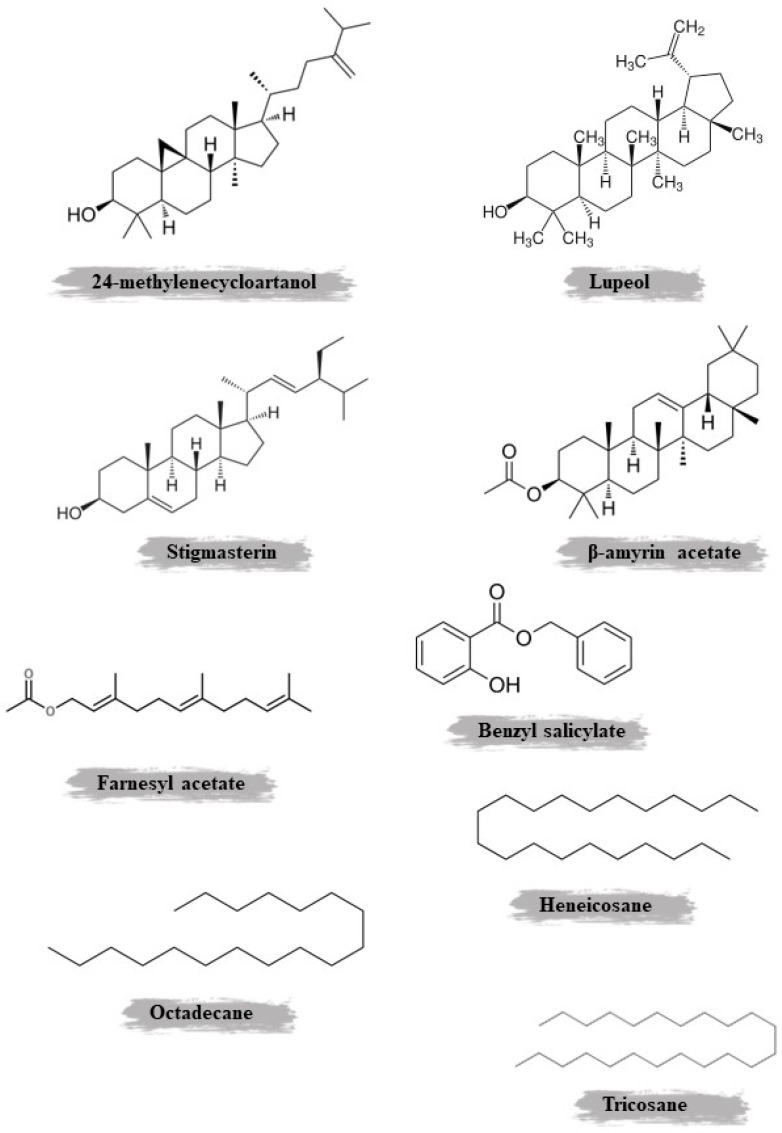
Chemical structure of molecules identified and isolated from *S. undulata*.

Harkati et al.’s study revealed that the volatile fraction of the roots of *S. undulata* ssp. deliciosa in Algeria predominantly contains hexadecanoic acid, n-tetradecanoic acid, 9-octadecenoic acid (the primary fatty acid in olive oil), and 9-hexadecenoic acid as key components [26]. Recently, it was reported that the aerial parts of this species are rich in sesquiterpenes and terpenoids [33], while the tubers of this plant are rich in terpenoids [32]. Figure 3 illustrates the chemical structure of important non-phenolic compounds identified and isolated from *S. undulata*.

According to the data of the present review, *S. undulata* is also distinguished by its essential oil and aromatic compounds, which contribute to its characteristic aroma and therapeutic potential. The essential oils extracted from *S. undulata* are known to contain a diverse array of bioactive constituents, including terpenoids, sesquiterpenes, and aromatic compounds. These essential oil components play a crucial role in the plant’s antimicrobial, antifungal, and anti-inflammatory properties, making *S. undulata* a valuable resource in traditional and modern medicine. Studies have investigated the ability of *S. undulata* essential oils to inhibit the growth of various pathogenic microorganisms, such as bacteria and fungi, as well as their potential to reduce inflammation.

The antimicrobial and anti-inflammatory activities of *S. undulata* essential oils support their use in natural remedies and pharmaceutical applications. Additionally, the pleasant scent of these oils makes them suitable for use in aromatherapy and cosmetic formulations, providing both health benefits and sensory experiences. The unique essential oil composition of *S. undulata* is not only responsible for its distinctive aroma but also serves as a chemical signature for the species within the Asteraceae family. Further research into the specific bioactive compounds and their mechanisms of action can contribute to a deeper understanding of the plant’s therapeutic potential and guide the development of novel natural products derived from *S. undulata* essential oils.

### 3.6. Pharmacological and Biological Activities

Natural products such as polyphenols, flavonoids, tannins, and isocoumarins, along with their glycosylated counterparts, have been implicated in several pharmacological and biological activities. Polyphenolic flavonoids and tannins exhibit highly potent anti-inflammatory activity by modulating inflammatory processes, inhibiting pro-inflammatory enzymes such as cyclooxygenase (COX) and lipoxygenase (LOX), and downregulating cytokines such as TNF-α and IL-6 [51]. In antidiabetic activity, these compounds can facilitate insulin secretion, decrease insulin resistance, enhance glucose uptake, and inhibit carbohydrate-hydrolyzing enzymes like α-glucosidase [52]. In anticancer activity, they can induce apoptosis, inhibit cell proliferation and angiogenesis, and modulate detoxification enzymes [53]. In addition to this, flavonoids and tannins are also involved in antihypertensive activity by induction of vasodilation via nitric oxide (NO) formation, angiotensin-converting enzyme (ACE) inhibition, and as calcium channel blockers [54]. Isocoumarins and their glycosylated derivatives also possess a broad spectrum of biological activities [55]. Some isocoumarins are anti-inflammatory, having inhibited the production of inflammatory mediators [56]. Some others possess antidiabetic activities such as α-glucosidase-inhibitory activity [57]. Moreover, isocoumarins were discovered to exhibit anticancer properties by mechanisms involving the induction of cell-cycle arrest and apoptosis in cancer cells [58]. Though not so well investigated for their direct antihypertensive action compared to flavonoids and tannins, certain derivatives of isocoumarins were found to have vasomodulatory activity [59]. Glycosylation of such naturally occurring compounds can often affect their solubility, bioavailability, and, hence, their pharmacological activities [60].

Table 3 provides a comprehensive outline of various biological (in vivo and in vitro) and pharmacological studies conducted on diverse extracts of *S. undulata*. The table includes essential data points such as the studied biological or pharmacological activity, the specific part of the plant targeted by the extract, the extraction technique employed, the dosage of the studied extract, the positive control, the inhibition percentage, and the primary outcomes observed in each study.

**Figure 4:**
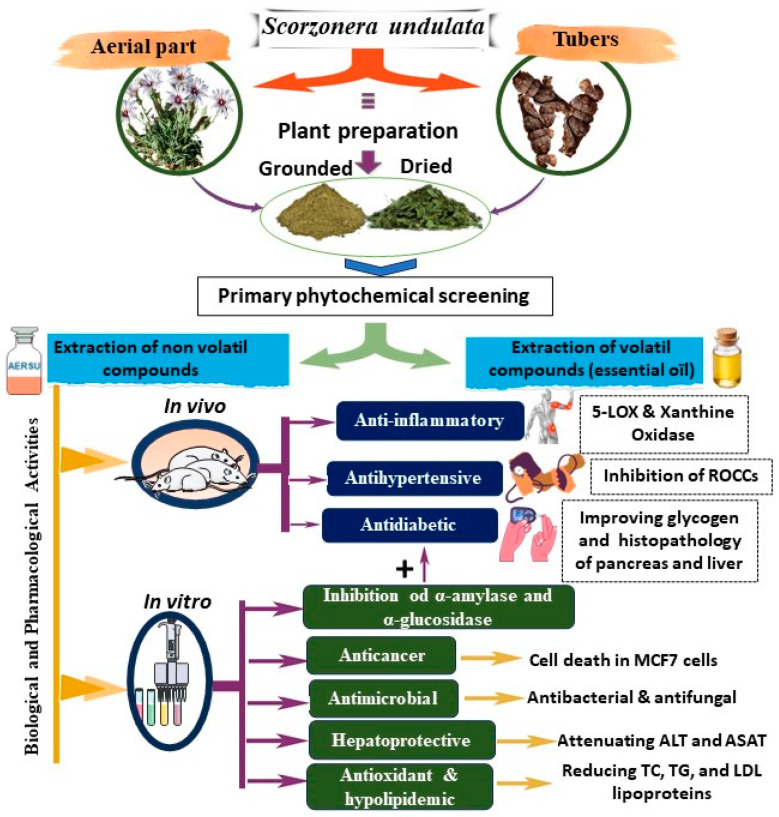
A schematic illustration of the main biological and pharmacological activities of *S. undulata*.

*S. undulata*, a medicinal plant native to various regions, including the southeast of Morocco, has been the subject of in vitro and in vivo biological research due to its wide range of biological and pharmacological properties. This plant is traditionally used in the form of different extracts, which have been found to possess diverse therapeutic effects. Our comprehensive literature review revealed that *S. undulata* extracts exhibit a broad spectrum of beneficial properties, making them a valuable resource in traditional medicine. These extracts have been reported to possess anti-inflammatory, anticancer, antidiabetic, antimicrobial, antihypertensive, cardioprotective, hepatoprotective, laxative, and hypolipidemic effects (Figure 4). The anti-inflammatory properties of *S. undulata* extracts can be beneficial in the management of various inflammatory conditions, while their anticancer effects have been demonstrated against multiple cancer cell lines, suggesting their potential as natural anticancer agents. The antidiabetic potential of these extracts has been attributed to their antihyperglycemic and hepatoprotective effects, which can be advantageous in the management of diabetes and its associated complications. Additionally, the antimicrobial activities of *S. undulata* extracts, including antibacterial and antifungal properties, indicate their potential as natural antimicrobial agents. Furthermore, the antihypertensive effects of these extracts have been reported, highlighting their potential in the management of hypertension. The cardioprotective properties of *S. undulata*, such as improved lipid profiles and protective effects on the heart, underscore its benefits for cardiovascular health. The hepatoprotective effects of these extracts have also been documented, suggesting their potential in the management of liver-related disorders. Additionally, *S. undulata* has traditionally been used as a laxative and has been found to have a positive impact on lipid metabolism, making it a potential hypolipidemic agent.

A recent study reported that the bioavailability and pharmacokinetics analyses of methanolic extracts from the roots and aerial parts of *S. undulata* showed that its compounds are biologically active. Most of these compounds adhere to Lipinski’s rule, exhibit low-to-moderate skin permeability, and possess acceptable bioavailability scores (BASs). These findings were further confirmed by bioavailability polygons based on the physicochemical properties of the compounds. While only p-coumaric acid and ferulic acid were found to permeate the blood–brain barrier (BBB), most of the identified compounds demonstrated high gastrointestinal absorption. The boiled egg model also supported these calculations: p-coumaric acid and ferulic acid fell within the yellow area, indicating BBB permeability, while the other compounds occupied white or grey areas, corresponding to high and low GI absorption, respectively. Additionally, most of the compounds do not act as substrates for P-glycoprotein (P-gp), suggesting no disruption in drug distribution. Safety assessments showed that none of the compounds inhibited the cytochrome P450 (CYP) isoforms tested, including CYP1A2, CYP2C19, CYP2C9, CYP2D6, and CYP3A4. Other compounds from this species also showed no inhibition of the studied CYPs [37].

#### 3.6.1. Antidiabetic Activity

In their 2020 study, Ajebli and colleagues demonstrated that both single and repeated oral doses of the aqueous extract derived from the aerial parts of *S. undulata* (SUAP), administered at a dosage of 20 mg/kg, resulted in significant reductions in blood glucose levels in rats induced with STZ. They further established that a single oral dose of SUAP, even at a high fixed dose of 3000 mg/kg body weight, did not exhibit any significant acute toxicological effects. This was evidenced by the fact that the oral LD50 of SUAP exceeded 3000 mg/kg body weight. Moreover, when SUAP was administered orally over a period of 15 days, it led to an increase in the glycogen content in the livers of diabetic rats. This treatment also improved the histopathological state of the liver and pancreas in the treated diabetic animals, and it ameliorated certain biochemical parameters, such as ALT and creatinine levels. However, it is important to note that SUAP did not have any effect on α-amylase activity. In addition to these findings, a preliminary phytochemical investigation revealed that the roots of *S. undulata* are rich in various phytochemicals, with a particular abundance of polyphenols. This suggests potential avenues for further research into the therapeutic properties of this plant [33].

The same authors investigated the antihyperglycemic potential of the aqueous extract from the roots and tubers of *S. undulata* (AERSU) in normal and diabetic rats. The researchers found that both single and repeated oral doses of AERSU at a dosage of 20 mg/kg resulted in a significant decrease in blood glucose levels in both normal and diabetic rats. Moreover, when AERSU was administered orally over a period of 15 days, it led to an increase in the glycogen content in the livers of both normal and diabetic rats. This treatment also inhibited α-amylase activity, which is noteworthy, as this enzyme plays a crucial role in carbohydrate digestion. By inhibiting α-amylase, AERSU may help slow down the absorption of glucose, contributing to improved blood glucose control. In addition to these metabolic effects, the treatment with AERSU improved the histological structure of the liver and pancreas in diabetic rats. This was accompanied by an improvement in certain biochemical parameters, such as ALT and AST levels, which are key indicators of liver function. These findings suggest that *S. undulata* extracts may have protective effects on the liver and pancreas, which are often affected in diabetes. Furthermore, a preliminary phytochemical investigation revealed that the roots of *S. undulata* are rich in various phytochemicals, with a particular abundance of polyphenols. Polyphenols are known for their antioxidant properties and have been associated with various health benefits, including improved glucose metabolism and reduced inflammation. These findings underscore the potential of *S. undulata* as a natural remedy for managing blood glucose levels and improving hepatic and pancreatic health in individuals with diabetes. The antihyperglycemic effects, coupled with the potential protective properties on vital organs, make *S. undulata* a promising candidate for further research and development as a complementary or alternative treatment for diabetes [32].

The comparison between the two previous studies—the first study assessed the antidiabetic activity of *S. undulata*’s aerial parts, while the second evaluated its tubers—underscores the different antidiabetic mechanisms of various components of *S. undulata*. The first study concentrated on the aerial parts and revealed considerable blood glucose reductions, liver glycogen increases, and histopathological improvements, with no influence on α-amylase activity, pointing toward a mechanism unrelated to carbohydrate-digesting enzymes. On the other hand, the second examined tuber extracts, which not only lowered blood glucose but also inhibited α-amylase, implicating another regulatory mechanism linked with carbohydrate metabolism. Protective effects on the liver and pancreas were confirmed in both studies; however, the contrasting biochemical responses, i.e., modulation of ALT and AST, imply that aerial and tuber extracts can act synergistically in an integrated treatment of diabetes. The results show that various components of the plant may have separate but complementary pathways, illustrating the medicinal diversity of *S. undulata* in regulating metabolism.

A recent study aimed to evaluate the antidiabetic and antioxidant effects of ethanolic extracts of *Scorzonera cinerea* leaves in diabetic rats (same model). The findings revealed that the dried extracts were more effective in inhibiting α-amylase, while frozen extracts better inhibited α-glucosidase. Both extracts reduced blood glucose and HbA1c levels, increased insulin, and improved liver antioxidant markers, while also counteracting oxidative stress indicators. Overall, *S. cinerea* shows promise as a dietary supplement with hypoglycemic and antioxidant properties that could benefit diabetes management [62]. Another antidiabetic activity study found that *S. sublanata* (another species of *Scorzonera* genus) extract produced the greatest effect in reducing blood glucose levels. When administered at a dose of 100 mg/kg, it significantly lowered blood glucose readings compared to the isotonic saline solution control group of diabetic animals. *S. cana* var. jacquiniana extract also noticeably decreased blood glucose after 4 h [63]. In the same respect, antidiabetic testing on the acetone extract of *Scorzonera phaeopappa* found that it exhibited twice the α-amylase enzyme inhibition of the drug acarbose. Against α-glucosidase, *S. phaeopappa* extract showed the strongest inhibitory activity, with an IC_50_ value of 6.3 mg/mL. A significant positive correlation was observed between the total phenol content and inhibition of α-glucosidase, indicating that higher phenol levels relate to greater α-glucosidase inhibition. Additionally, a significant positive correlation was observed between the total terpene content and inhibition of α-glucosidase, suggesting that higher terpene amounts produce increased α-glucosidase inhibition [64]. Interestingly, the extract from the aerial parts of *S. latifolia* demonstrated hypoglycemic effects. Additionally, compounds such as swertisin, 7-O-methyl-isoorientin, and hydrangenol-8-O-β-glucoside identified in this species show promise for their potential hypoglycemic properties [65].

In comparison with other *Scorzonera* species, *S. undulata* exerts unique antidiabetic characteristics, and the aerial and tuber extracts influence the metabolic pathways differently. In contrast to *S. cinerea* or *S. phaeopappa*, which exhibited potent α-glucosidase inhibition, *S. undulata* aerial extracts did not affect α-amylase, while its tuber extract was a potent α-amylase inhibitor. This indicates that *S. undulata* possesses a distinct dual strategy of glucose regulation, with direct reduction in blood glucose levels through its aerial parts and enzymatic modulation from its tubers. Additionally, its high content of polyphenols is congruent with the antioxidant and hypoglycemic properties observed in *S. latifolia* and *S. sublanata*, hence its therapeutic significance in the genus. The reported protective effect on liver and pancreas functions also underscores its value as a potent drug candidate for the treatment of diabetes.

#### 3.6.2. Anticancer Activity

The in vitro anti-proliferative activity of *S. undulata* extracts was evaluated using the MTT assay on MCF7 breast cancer cells. The results revealed a dose-dependent inhibitory effect on cell growth for both the root (RSU) and aerial (ASU) extracts. At 24 h of incubation, the concentration required to inhibit 50% of cell proliferation (IC_50_) was 4.22 ± 0.06 mg/mL for the RSU extract and 5.89 ± 0.08 mg/mL for the ASU extract. When the cells were incubated with increasing concentrations of the extracts for 48 h, the IC_50_ values decreased to 2.89 ± 0.15 mg/mL for RSU and 4.42 ± 0.015 mg/mL for ASU. Microscopic examination of the treated cells revealed a significant decrease in cell density following the treatment with both the RSU and ASU extracts. The microscopic slides also showed evidence of dead cells, apoptotic bodies, cellular shape deformation, and shrinkage, after both one and two days of incubation with the *S. undulata* extracts. These findings demonstrate the dose-dependent anti-proliferative activity of *S. undulata* extracts against MCF7 breast cancer cells. The observed cytotoxic effects, including the induction of apoptosis and morphological changes, suggest that the extracts from this medicinal plant possess potent anticancer properties that warrant further investigation. Generally, the ability of *S. undulata* extracts to inhibit the growth of MCF7 breast cancer cells in a concentration-dependent manner highlights their potential as a source of natural compounds with therapeutic applications in the management of breast cancer. Further research is needed to elucidate the specific phytochemicals responsible for the observed anti-proliferative effects, and to explore the underlying mechanisms of action [37]. According to microscopic analysis, the cytotoxicity of *S. undulata* is believed to occur through cell lysis and an increased apoptosis pathway. The confirmation of apoptosis was achieved using flow cytometry with the JC-1 kit, which detects changes in mitochondrial transmembrane potential indicating cell death [37]. Cell death is a basic biological process that is seen in a wide range of biological situations, ranging from the development of organisms to the regulation of tissue homeostasis. The function of cells can be halted by discrete mechanisms, and the description of these processes is relevant to an insight into the regulation of cells’ life and death [66]. Cell lysis and cell disruption involve the breakdown of cells through either self-induced processes or external damage to the outer membrane [67]. This process can occur under various conditions, and over time, numerous techniques have been developed to facilitate cell disruption. Naturally, cell lysis happens when cells undergo apoptosis, aiding in detoxification and the removal of unwanted cells [68], whereas apoptosis resembles a graceful ballet of cellular death, embodying a precisely regulated form of programmed cell death (PCD). It unfolds in a controlled way, independent of external triggers, highlighting the cell’s internal regulatory mechanisms [69]. This process is initiated by biochemical signals encoded in the cell’s DNA, guiding a specific pathway that leads to its self-destruction [70]. Another recent investigation provides new insights into the cytotoxic, apoptotic, and genotoxic effects of *S. pygmaea* extract. The extract induced apoptosis in both cancerous and non-cancerous cell lines, suggesting its potential for cancer prevention in nutraceuticals. It also caused DNA damage, with the extent varying based on exposure duration and cell type, reflecting complex interactions between its constituents and cellular mechanisms, influenced by changes in cell-cycle regulator expression. Future research should evaluate *S. pygmaea* as a potent antioxidant source, particularly rich in chlorogenic acid, while balancing its health-promoting properties against potential genotoxic risks, highlighting its promising role in the food industry and human health [71].

According to the investigations of Lendzion et al. (2022), in biological assays, D-chiro-inositol, β-sitosterol, and methyl 3,4-dimethoxycinnamate (isolated from *Scorzonera hispanica* seeds) demonstrated cytotoxic activity against the MCF-7 human mammary carcinoma cell line by inhibiting the PI3K/Akt and ERK 1/2 signaling pathways. Additionally, the compounds SH1, SH4, and SH11 were found to affect the expression of proteins involved in apoptosis and autophagy. Their inhibitory effects on IL-8 expression may contribute to the suppression of angiogenesis and tumor metastasis [44].

Unlike other *Scorzonera* species, *S. undulata* is reported to possess excellent anti-proliferative activity against MCF7 breast cancer cells, with dose-dependent inhibition like *S. pygmaea*, but distinct in apoptosis induction. Unlike *S. hispanica*, which inhibits the PI3K/Akt and ERK 1/2 pathways, *S. undulata* predominantly induces apoptosis by mitochondrial membrane potential loss, as evidenced by JC-1 staining. Its cytotoxicity profile demonstrates a novel mechanism that includes both cell lysis and apoptotic body formation, confirming its potential as an anticancer agent. Comparative studies are needed to ascertain whether its polyphenol-rich content is responsible for its novel anticancer activity.

#### 3.6.3. Anti-Inflammatory Effects

A comprehensive study was conducted to evaluate the wound-healing and anti-inflammatory effects of the aerial parts and roots of various *Scorzonera* species found in Turkey. The researchers focused on *S. acuminata*, *S. cana* var. alpina, *S. cana* var. jacquiniana, *S. cana* var. radicosa, *S. eriophora*, *S. laciniata* ssp. laciniata, *S. suberosa* ssp. suberosa, and *S. sublanata* to clarify the traditional usage of these plants in Turkish and European folk medicine [30]. It is well established that certain *Scorzonera* species have been traditionally used for wound-healing purposes [72]. To assess the wound-healing effect, the researchers employed linear incision and circular excision experimental wound models, followed by histopathological analysis. They also evaluated the hydroxyproline content of the treated tissues, which is an indicator of collagen synthesis and wound healing. Furthermore, the extracts were screened for anti-hyaluronidase activity, as this enzyme plays a crucial role in inflammation. To evaluate the anti-inflammatory activity, the researchers used the acetic acid-induced increase in capillary permeability test. The results of the study revealed that the 20% aqueous methanol extracts of the aerial parts of *S. cana* var. jacquiniana and *S. eriophora* were effective in the wound-healing and anti-inflammatory activity test models. The histopathological examination supported the findings from the linear incision and circular excision wound models. These findings provide scientific validation for the traditional use of certain *Scorzonera* species in wound-healing and anti-inflammatory applications [73]. In 2018, Bahadır-Acıkara and colleagues conducted a study that discovered that n-hexane extracts from the roots and above-ground parts of eleven different *Scorzonera* species (namely, *S. acuminata*, *S. cinerea*, *S. eriophora*, *S. incisa*, *S. latifolia*, *S. mirabilis*, *S. mollis* ssp. szowitsii, *S. parviflora*, *S. suberosa* ssp. suberosa, and *S. tomentosa*) were rich in triterpenes. These triterpenes included significant quantities of taraxasteryl acetate, lupeol, and lupeol acetate [74]. Generally, the root extracts were found to be significantly more abundant in the analyzed triterpenes. However, lupeol was an exception. The concentration of lupeol in the aerial parts of *S. incisa*, *S. latifolia*, *S. mirabilis*, *S. parviflora*, and *S. suberosa* was about three to seven times greater than in the root extracts, with a range of approximately 0.9–1.5 mg/g [13]. These findings align with previous observations that linked lupeol to anti-inflammatory and analgesic effects [75,76].

Bahadir et al. (2015) provided evidence for the anti-inflammatory potential of certain *Scorzonera* extracts. Using an in vitro assay, the extracts inhibited the production of TNF-α and IL-1β, pro-inflammatory molecules, in LPS-stimulated THP-1 macrophages. This effect was further supported by the observed suppression of NF-κB activation, a key pathway in inflammation. Interestingly, while several compounds isolated from these extracts were tested for their ability to inhibit TNF-α and IL-1β production, none displayed significant activity. This finding highlights the need for further research to pinpoint the specific active components responsible for the observed anti-inflammatory effects of the *Scorzonera* extracts [72].

Both previous studies together suggest that different species of *Scorzonera* can be utilized to treat wounds and inhibit inflammation. They have the same action of stimulating collagen synthesis and inhibiting inflammation-causing enzymes. Both studies confirm the application of *Scorzonera* in folk medicine, especially in wound- and inflammation-related ailments, yet they do differ considerably in what they concentrate on: one highlights methanol extracts of the above-ground parts and their activity in wound-healing models, while the other points out that triterpenes are present in the root and above-ground extracts, namely, lupeol, which is associated with reducing inflammation. Some extracts have shown that they are able to directly suppress pro-inflammatory molecules like TNF-α and IL-1β, but further research needs to be conducted to determine the actual active constituents exerting such effects. This variation shows just how complex and useful *Scorzonera* species could be in medicine.

TNF-α and IL-1β have long been recognized as key drivers of inflammation in diseases like rheumatoid arthritis, Crohn’s disease, and ulcerative colitis [77]. They have been classically thought of as two of the most powerful pro-inflammatory cytokines and are known to play a pivotal role in inducing and maintaining inflammatory responses in an array of diseases and pathological situations [78]. These cytokines are produced by mononuclear leukocytes in response to various stimuli, including endotoxins and lipopolysaccharides [79]. Elevated levels of TNF-α and IL-1 are characteristic features of many inflammatory diseases. TNF-α is the prototype of a large cytokine family known as the TNF ligand family. It is primarily produced as a type II transmembrane protein that forms stable homotrimers. From this membrane-bound form, the soluble homotrimeric cytokine (soluble TNF-α, or sTNF-α) is released through proteolytic cleavage by the metalloprotease TNF-α-converting enzyme (TACE). The soluble 51 kDa trimeric TNF tends to dissociate at concentrations below the nanomolar range, which results in a loss of its biological activity. Members of the TNF ligand family carry out their biological effects by binding to their specific membrane receptors, collectively known as the TNF receptor (TNFR) family [80]. The production and activity of IL-1β are tightly regulated at multiple stages, including transcription, translation, cleavage, and cellular secretion [81]. In macrophages, for example, IL-1β activation requires two distinct, sequential intracellular signals that promote its transcription and translation [82]. Both soluble IL-1β and IL-1α trigger cellular responses by binding to the transmembrane receptors IL-1R1 or IL-1R3. Additionally, the IL-1 receptor antagonist (IL-1ra) binds to IL-1R1 with similar specificity and affinity but does not activate the receptor or initiate downstream signaling [83]. The transcription factor NF-κB controls prominent aspects of innate and adaptive immunity and acts as a master regulator of inflammation. NF-κB stimulates the expression of numerous pro-inflammatory genes, including cytokines and chemokines, and modulates the function of inflammasomes. Moreover, NF-κB is also essential for the survival, activation, and differentiation of innate immune cells and inflammatory T cells. Aberrant NF-κB activation has been implicated in the etiopathogenesis of diverse inflammatory diseases. NF-κB’s activation and function will be addressed here as part of a review on inflammation and current therapeutic advances in NF-κB inhibition [84].

In comparison to other species of *Scorzonera* tested for wound-healing and anti-inflammatory activities, *S. undulata* has demonstrated unique pharmacological activities, extending beyond mere inflammation control. Whereas other species, such as *S. cana* var. jacquiniana and *S. eriophora*, exhibited wound-healing activity via hydroxyproline-stimulated collagen synthesis, *S. undulata* has demonstrated systemic effects, including antidiabetic activity and cytotoxicity. Furthermore, the reported presence of polyphenols and potential bioactive compounds in *S. undulata* overlaps with *S. suberosa* and *S. latifolia* triterpene-rich extracts, but further research is needed to determine whether *S. undulata* also shares similar mechanisms of inflammation inhibition. Its broader therapeutic scope suggests a sophisticated medicinal application in the *Scorzonera* genus.

#### 3.6.4. Antimicrobial Activity

Ben Abdelkader et al. (2010) studied the antibacterial and antifungal effects of the extracts (butanolic, ethyl acetate, petroleum ether, and product H2) from two plants (*Rhaponticum acaule* L. and *S. undulata*) in vitro, using disc diffusion and liquid dilution techniques. The findings of this investigation revealed that the ethyl acetate extract obtained from the aerial parts of *S. undulata* exhibited antibacterial activity against five bacterial strains (*P. aeruginosa*, *S. aureus*, *E. fecalis*, *C. freundeï*, and *P. mirabilis*). The minimum inhibitory concentration (MIC) of this extract ranged from 1000 to 2000 µg/mL. Notably, the petroleum ether extract from the same plant material only showed antibacterial activity against three strains (*P. aeruginosa*, *S. aureus*, and *C. freundeï*). Moreover, these outcomes were found to be consistent with the results obtained using the disc diffusion method. Furthermore, the findings of this study revealed that both the ethyl acetate and petroleum ether extracts displayed antibacterial activity against three strains of bacteria: *Staphylococcus aureus*, *Enterococcus faecalis*, and *Citrobacter freundii*. However, the petroleum ether extract demonstrated greater potency against the Gram-positive bacterium *S. aureus*, with a minimum inhibitory concentration (MIC) of 500 µg/mL, compared to the ethyl acetate extract. Interestingly, the researchers observed that all of the tested fractions exhibited stronger antibacterial activity against the Gram-positive bacteria (*S. aureus* and *E. faecalis*) compared to the Gram-negative bacterium *C. freundii* [24]. This suggests that the phytochemical constituents present in the *S. undulata* root extracts may be more effective in inhibiting the growth of Gram-positive bacterial strains [85]. Interestingly, the enhanced antibacterial activity of the petroleum ether extract against *S. aureus*, a common pathogen associated with various skin and soft tissue infections, is particularly noteworthy. This finding indicates the potential of *S. undulata* as a natural source of antimicrobial compounds that could be harnessed for the development of novel therapeutic agents or as adjuncts to conventional antibiotics. The petroleum ether extract from the aerial part of *S. undulata* significantly hindered the growth of three fungi: *A. fumigatus* (48.21%), *T. rubrum* (58.92%), and *S. brevicaulis* (53.57%). It also exhibited excellent inhibitory activity against *M. canis* (78.57%) with MIC values ranging from 500 to 750 µg/mL. The ethyl acetate extract from the aerial part also strongly inhibited *M. canis*, with an inhibition percentage of 64.24% and an MIC of 700 µg/mL. The root ethyl acetate extract showed minimal inhibition (3.57%) against *A. fumigatus*, while *M. canis* (46.42%) and *T. rubrum* (46.42%) were moderately inhibited by the root petroleum ether extract, with an MIC of 800 µg/mL. However, none of the extracts from the two species showed any activity against *C. albicans* and *C. neoformans* [24]. Likewise, Boussaada and their team conducted an experiment on the antibacterial activity of *S. undulata* subsp. deliciosa oil. They used paper disc diffusion and dilution techniques for the evaluation. The test was carried out against six chosen species of bacteria, which included Gram-positive and Gram-negative types: *Staphylococcus aureus*, *Staphylococcus epidermidis*, *Escherichia coli*, *Pseudomonas aeruginosa*, *Micrococcus luteus*, and *Salmonella typhimurium* from a hospital source. The oil demonstrated a range of antibacterial activities from moderate to significant against all Gram-positive cocci and Gram-negative rod bacteria, with the exception of *Pseudomonas aeruginosa*. The oil from subsp. *deliciosa* showed greater effectiveness against *Staphylococcus aureus* and *Micrococcus luteus*, inhibiting their growth significantly at lower concentrations (MIC: 0.5 mg/mL and MBC: 0.8 mg/mL). However, its effectiveness was reduced against other microorganisms, such as *Staphylococcus epidermidis*, *Salmonella typhimurium*, and *Escherichia coli* [11]. Experiments on the antifungal activity of the same extract were carried out against four types of phytopathogenic fungi. These fungi were sourced from the Phytopathology Laboratory at the Regional Center for Agricultural Research and Development in the eastern part of Chott-Mariem, Sousse, Tunisia. The fungi tested were *Fusarium oxysporum*, *Aspergillus niger*, *Penicillium* sp., and *Alternaria* sp. The disc diffusion technique was employed for the antifungal tests. The findings of this study revealed no antifungal activity against the four pathogenic fungi screened [11].

Many researchers’ studies have found that *Scorzonera* extracts exhibit varying levels of antimicrobial action against both Gram-positive and Gram-negative microorganisms, although the antifungal effect is either low or nonexistent [86,87]. In addition, plant extracts from certain species of the *Scorzonera* genus (namely, *Scorzonera suberosa* (C. Koch), *Scorzonera latifolia* (Fisch. and Mey.), and *Scorzonera laciniata* (L.)) demonstrated varying degrees of antimicrobial activity against both Gram-positive and Gram-negative bacteria. However, only the *S. suberosa* extract exhibited an antifungal effect. As a result, it was inferred that the antimicrobial activity of the samples tested in the study surpassed their antifungal activity. This observation aligns with the results reported in the existing literature [88]. Recently (2023), Güçlü and colleagues carried out a study on the antimicrobial activity of 80% ethanol extracts from the leaves of *Scorzonera* tomentosa. The activity was tested against *C. albicans*, *B. cereus*, *P. aeruginosa*, *E. coli*, and *S. aureus* using broth microdilution analyses. The concentration range used was from 0.312 to over 2.5 mg/mL. The findings indicated that the extract exhibited only mild antimicrobial effects on the strains tested [89]. Ak et al. (2022) investigated the skin-protective and antimicrobial effects of extracts from *S. hieraciifolia*, *S. hispanica*, and *S. tomentosa*. The extracts showed antimicrobial activity against several bacterial and fungal strains, and they protected mouse skin from hydrogen peroxide damage by reducing L-dopa and prostaglandin E2 levels (indicators of tyrosinase activity and inflammation). These findings suggest that *Scorzonera* species, potentially due to flavonoids like rutin, may be useful in managing inflammatory and infectious skin conditions [90].

In fact, phytochemical investigations of various parts of *S. undulata* have revealed a rich array of bioactive compounds, including polyphenols (flavonoids and tannins), terpenoids, fatty acids, and essential oils. These phytochemicals are believed to play a crucial role in the observed biological properties of this medicinal plant. Generally, the diverse pharmacological properties of *S. undulata*, coupled with its rich phytochemical profile, underscore its potential as a natural therapeutic agent. Further research is necessary to fully elucidate the mechanisms underlying these properties, and to explore the potential of *S. undulata* in the development of new therapeutic strategies for a wide range of health conditions. Interestingly, plant-derived antimicrobials offer promising benefits. They have the potential to treat serious illnesses while avoiding many of the adverse effects associated with synthetic antimicrobials. The therapeutic efficacy of plant materials is often attributed to the synergistic interaction of various compounds within the plant [91]. Antimicrobial agents act by a variety of mechanisms against critical microbial processes. Another major one acts by inhibiting protein synthesis by binding to bacterial ribosomes (30S or 50S subunits), e.g., aminoglycosides, tetracyclines, macrolides, and chloramphenicol, leading to the inhibition of translation [92]. Additionally, certain agents suppress the synthesis of nucleic acids by disrupting DNA replication or RNA transcription [93]. Such varied mechanisms enable selective toxicity to microbes through the exploitation of differences in cellular processes and structures from host cells.

In comparison with other *Scorzonera* species, *S. undulata* displays substantial antibacterial properties, mainly against *S. aureus*, *P. aeruginosa*, and *E. faecalis*, with ethyl acetate and petroleum ether extracts demonstrating varying effectiveness. Though lacking inhibition against *C. albicans* and *C. neoformans*, *S. undulata* showed selective antifungal activity, most notably against *M. canis*, unlike *S. suberosa*, which showed antifungal effects. Furthermore, although antimicrobial properties across the genus vary, *S. undulata* subsp. deliciosa showed great antibacterial potency at lower concentrations, especially against Gram-positive bacteria, implying possible therapeutic uses. This emphasizes its unique antimicrobial profile, which calls for more research of its phytochemical components in order to better grasp its modes of action.

#### 3.6.5. Hypolipidemic Activity

A recent study indicates that the water-based extract from the tubers of *S. undulata* possesses a strong capacity to enhance blood lipoprotein lipids. It also exhibits a noteworthy anti-atherogenic effect and bolsters the antioxidant system in both healthy and diabetic rats. The antioxidants found in the raw extract could partially account for the plant tubers’ lipid-lowering effects. The findings of this study indicate that further investigations are needed to identify and analyze the active compound(s) in the raw extract that contribute to its lipid-reducing and antioxidant properties, and to utilize these compounds in bioassay-guided experiments. Moreover, ongoing advanced pharmacological and biochemical studies aim to clarify the mechanisms that underpin these properties demonstrated by *S. undulata* tubers [61].

### 3.7. Toxicity

Our literature review revealed that only two toxicity studies on *S. undulata* had been undertaken, both conducted by our research team in 2020 and 2021. One assessed the acute toxicity of the aqueous extract from the plant’s aerial parts, while the other evaluated the aqueous tuber extract. The primary study’s results showed that single oral administrations of the aerial extract (AESU) at doses of 1000, 2000, and 3000 mg/kg body weight over 14 days produced no signs of toxicity, changes in body weight, or mortality in the treated animals. However, AESU significantly increased the treated animals’ body weight. *S. undulata* ssp. deliciosa roots are traditionally consumed as food in Morocco and are considered to be edible plants. The present study supports the safety of using this plant as both a food and traditional medicine. Additionally, the results align with those of other reports showing AESU’s positive effects on liver function biomarkers (ALT, AST) and kidney markers (creatinine, urea). Improvement was also observed in histopathological analyses of the liver, further corroborating AESU’s tolerability [33]. Similarly, for the aqueous tuber extract, this study found that no toxicity signs, mortality, or behavioral changes were observed in male and female rats administered either distilled water (control) or *S. undulata* tuber extract (TESU) at doses of 1000, 2000, or 3000 mg/kg body weight in the acute toxicity test. When TESU was administered at these doses daily for 14 days, rats remained alert, without motor or neurological issues. No adverse effects were seen relating to the gastrointestinal, respiratory, or locomotor systems for either sex. Furthermore, TESU had a positive impact on body weight changes across groups for both male and female rats. The extract was well tolerated at all test doses based on the lack of adverse outcomes through clinical observations and weight measurements [32].

While the available data provide preliminary evidence for the tolerability of *S. undulata* extracts, additional toxicological studies are still warranted to fully evaluate its safety profile. Sub-chronic and chronic toxicity tests involving repeated exposure over longer durations would help assess the potential risks from long-term consumption. Investigating the toxicity of other *S. undulata* extracts derived from different plant parts or extraction methods could uncover any variations. Using increased dosage levels in toxicity studies may better define the acceptable safe limits. Experiments with other animal species as models, such as dogs, would facilitate extrapolation to human health risks. Comprehensive chemical profiling of extracts can aid in identifying constituents requiring deeper toxicological evaluation. Overall, further research incorporating a variety of study designs and testing paradigms would strengthen confidence in the favorable safety margin suggested so far for this traditionally used medicinal plant. Continued investigation remains an important undertaking prior to any clinical therapeutic application.

Recent studies have reported that flavonoids from *S. austriaca* exhibit hepatoprotective effects through several mechanisms. They reduce the levels of liver injury markers such as AST, ALT, LDH, and MDA, while simultaneously increasing the levels of antioxidant enzymes like SOD and GSH-Px. FSA also inhibits inflammation by lowering the levels of TNF-α and IL-6, which it achieves by inactivating the TLR4/NF-κB signaling pathway. Additionally, FSA promotes autophagy, as evidenced by an increased LC3B-II/I ratio and decreased p62 protein levels [94].

### 3.8. Antioxidant Abilities

Table 4 presents data on the antioxidant activity of different parts of a plant, including tubers, leaves, and flowers. In this table, we present a comprehensive analysis of various antioxidant studies conducted on different extracts of *S. undulata*. The analysis focuses on several key aspects, including the extraction technique employed, the specific solvent utilized for extraction, the concentration used for assessing the antioxidant activity, the IC_50_ value obtained, the positive control employed in the antioxidant assay, and the primary outcomes derived from the assay. This table provides a structured overview of the antioxidant studies on various *S. undulata* extracts, offering insights into the methodologies, results, and comparisons in antioxidant activity.

Antioxidants help protect our cells from oxidative damage caused by free radicals. These harmful molecules can lead to various health issues, including aging, inflammation, and chronic diseases. By scavenging free radicals, antioxidants contribute to our overall well-being and health protection. Moreover, many chronic conditions, such as cardiovascular diseases, cancer, and neurodegenerative disorders, are linked to oxidative stress [96]. Plant extracts rich in antioxidants can mitigate this stress, potentially reducing the risk of such diseases, thereby playing a crucial role in disease prevention. Antioxidants also contribute to maintaining healthy skin [97]. They combat oxidative stress caused by UV radiation, pollution, and other environmental factors. Plant-based antioxidants can help with skin repair, collagen synthesis, and promoting a youthful appearance. Furthermore, inflammation is a common underlying factor in various health problems. Antioxidant-rich plant extracts possess anti-inflammatory, anti-proliferative, anticancer, antidiabetic, and cardioprotective properties, helping to manage inflammation and associated conditions. Interestingly, recent research suggests that plant extracts with antioxidant activity can influence the gut microbiota composition. A balanced gut microbiome is essential for overall health, immunity, and digestion, underscoring the importance of antioxidants for gut health [98].

Table 4 shows that antioxidant activity was measured using various assays, such as DPPH scavenging, CUPRAC, FRAP, and ABTS. The results show that the leaves and ethanolic extracts generally exhibited the highest antioxidant activity. In contrast, the tubers had lower antioxidant activity compared to the aerial parts, likely due to their role as storage organs containing compounds like inulin. Interestingly, the methanolic extracts of tubers showed the highest scavenging activity against radicals, with effective IC_50_ values. Additionally, the aerial parts had a higher potential to scavenge DPPH and higher total antioxidant activity compared to the tubers. The table also highlights the importance of the extraction method and solvent choice in determining the antioxidant potential of the plant parts. The data suggest a positive correlation between the antioxidant activity and the total polyphenolic content (TPC) of the plant parts, particularly in the leaves. Furthermore, the results are compared to standard antioxidant controls, such as vitamin C and BHT, which were used as references. The aqueous extracts of tubers (decoctions) also possessed potent antioxidant activity. Furthermore, the data of this review provide a comprehensive understanding of the antioxidant properties of different parts of the plant, demonstrating the influence of extraction methods and solvents on the observed activities.

Recent studies have highlighted intriguing antioxidant activity through various methodologies across multiple *Scorzonera* species. These findings reveal the diverse antioxidative potential present within the *Scorzonera* genus, offering valuable insights into the bioactive properties of these plants. Such research not only underscores the significance of antioxidant compounds in *Scorzonera* species but also paves the way for further exploration into their therapeutic applications and potential health benefits [99]. The antioxidant capabilities of the pure compounds from *S. papposa* and similar derivatives extracted from another *Scorzonera* species (*S. judaica* Eig.) were assessed. The relative antioxidant capacity index (RACI) was utilized as a comprehensive approach to compare the antioxidant properties measured through various chemical techniques [100].

Generally, exploring the antioxidant potential of plant extracts not only enhances our understanding of natural compounds but also offers promising avenues for preventive and therapeutic applications, addressing various aspects of human health and well-being.

It is important to acknowledge that the antioxidant activities evaluated for the extracts of this species have been predominantly conducted through in vitro assays, as indicated in the existing literature. However, it is noteworthy that there is a lack of in vivo studies in the current review regarding these antioxidant properties. It is crucial to emphasize that in vivo antioxidant assessments play a vital role in providing significant insights and valuable data that address a broader range of biological and physiological inquiries. The comparison of in vivo and in vitro antioxidant activities is an important consideration when evaluating the therapeutic potential of natural extracts and compounds. While in vitro antioxidant assays provide valuable information about the direct free radical scavenging and reducing capabilities of a sample, they do not fully capture the complex interactions and bioavailability that occur in the living organism. In vivo antioxidant studies are crucial because they assess the ability of the test substance to exert antioxidant effects within the physiological context of a living system [101]. This includes factors such as absorption, distribution, metabolism, and excretion, which can significantly impact the bioactivity of the antioxidants [102]. Furthermore, in vivo models can reveal the potential for the antioxidants to modulate endogenous antioxidant systems and influence relevant biomarkers of oxidative stress and inflammation [103].

By comparing the in vitro and in vivo antioxidant activities of a natural extract or compound, researchers can gain a more comprehensive understanding of its therapeutic potential. Discrepancies between the two types of assays may indicate the need for further optimization of the delivery system, formulation, or dosage to enhance the bioavailability and in vivo efficacy of the antioxidants [104]. Ultimately, the integration of both in vitro and in vivo data is essential for the development of effective, natural-based antioxidant interventions with potential applications in various health domains.

Therefore, further exploration and incorporation of in vivo studies could offer a more comprehensive understanding of the potential impact and effectiveness of these extracts as antioxidants.

### 3.9. Critical Assessment and Limitations

This comprehensive review delves into an in-depth analysis of available data from various sources to provide valuable insights into *S. undulata*. By thoroughly utilizing important literature on the subject, this review aims to offer a holistic overview of this medicinal plant. However, it is essential to critically discuss various aspects and evaluate limitations to enhance the depth of this bibliographic study.

One key point that necessitates critical examination is the limited number of studies that have been conducted on *S. undulata*. This gap indicates a significant area where further research and exploration are needed to expand our understanding of the plant’s characteristics and potential benefits. This review highlights the need for more extensive investigations to uncover the full breadth of *S. undulata*’s phytochemical composition, biological activities, and therapeutic applications. Furthermore, extracts of *S. undulata* showed modest and variable antifungal activity, and this may restrict their application as drugs, particularly for resistant organisms. This variability may necessitate higher dosing, which would also increase the potential for toxicity [105]. To address these limitations, future research should focus on identifying and optimizing bioactive antifungal compounds, combining extracts with other antifungal agents for synergistic effects, improving extraction and cultivation methods, and employing biotechnological approaches to enhance the production of antifungal compounds. Moreover, rigorous testing against resistant strains and understanding of resistance mechanisms are crucial for guiding the development of more potent derivatives [106]. Furthermore, the inconsistent performance of *S. undulata* extracts in medicinal applications is due to variations in extraction methods, plant variability, and environmental conditions, which affect the concentration and profile of bioactive compounds. Additionally, the specific bioactive constituents responsible for its medicinal properties, particularly antifungal activity, remain largely unidentified [107]. To address these issues, researchers should implement standardized extraction protocols, control the cultivation conditions, and use advanced analytical techniques. Further strategies include bioassay-guided fractionation and in silico modeling to identify and predict the activity of key compounds. Interdisciplinary collaboration is crucial for optimizing these processes and enabling the development of reliable *S. undulata*-based pharmaceuticals.

Another crucial aspect to address is the absence of clinical trials focusing on the extracts derived from this plant. The reliance on preclinical and in vitro studies conducted on animal models highlights the need for more rigorous human trials to validate the efficacy and safety of these extracts for medicinal use. Bridging this gap is essential to translate the promising findings from laboratory studies into practical applications for human health. Furthermore, this review highlights a lack of studies investigating the biological and pharmacological properties of the individual molecules isolated from *S. undulata*. Delving into the specific compounds present in the plant could provide valuable insights into their potential therapeutic applications and mechanisms of action. Identifying and characterizing the bioactive constituents could lead to the development of more targeted and effective natural-based interventions. Lastly, there is a notable deficiency in exploring the mechanisms of action underlying the biological studies conducted on various extracts of *S. undulata*. Understanding how these extracts interact at a molecular level could offer valuable information for future research and the development of potential pharmaceutical applications. Elucidating the specific pathways and targets modulated by *S. undulata* could shed light on its therapeutic potential and guide the design of more effective formulations.

Commonly, while this review provides a comprehensive overview of *S. undulata* based on the existing literature, it also emphasizes the need for further research to address the identified gaps and limitations. By critically evaluating these points and conducting more exhaustive studies, researchers can deepen their knowledge and potentially unlock the full therapeutic potential of this intriguing plant species. Addressing the limitations highlighted in this review can pave the way for more impactful discoveries and the development of innovative, natural-based solutions for various health conditions.

The importance of dose-escalation studies for antidiabetic treatments is in defining the optimal therapeutic range, where efficacy is achieved with minimal adverse effects. These studies often use adaptive designs to step-refine dosing approaches in accordance with patient compliance, enhancing the precision of the drug development process. Acteoside, as a nutraceutical and functional food ingredient, has great potential owing to its antioxidant and anti-inflammatory activities, which can improve metabolic health and aid in the management of diabetes [108,109]. At the same time, the sustainability of *S. undulata* is affected by the combination of habitat destruction and insufficient cultivation activities. Other forms of habitat destruction include controlled breeding of the species, which supplies the market but does not protect the environment, thereby putting the species at risk of being exploited [110].

## 4. Conclusions

*S. undulata*, a plant found throughout the Mediterranean basin (Morocco, Algeria, Libya, Tunisia, and Turkey), boasts a rich history of traditional medicinal uses and a fascinating phytochemical profile. This review delves into these aspects, revealing its distribution across Morocco, particularly in the Daraa-Tafilalet (Boudnib) and Tata regions. Traditionally, *S. undulata* has been employed to address a wide range of ailments, including diabetes, constipation, diarrhea, and snake bites. Additionally, it is known for its diuretic, emollient, sudorific, depurative, and thirst-quenching properties.

Scientific exploration has confirmed the plant’s potential. Phytochemical analysis has identified a wealth of bioactive compounds within *S. undulata*, including phenolics, flavonoids, tannins, and various other categories. Specific examples include apigenin-7-glucoside, gallic acid, luteolin-7-glucoside, and p-coumaric acid. In vitro and in vivo studies further support its traditional uses. Extracts of *S. undulata* (methanolic, aqueous, butanolic, ethyl acetate, and petroleum ether) have exhibited anti-inflammatory, antidiabetic, anticancer, antibacterial, antifungal, antihypertensive, antilipidemic, and antioxidant properties. This impressive array of bioactivities aligns perfectly with the ethnobotanical applications of *S. undulata* in traditional medicine. Moreover, the abundance of antioxidants, anti-inflammatories, and other bioactive compounds positions *S. undulata* as a promising candidate for development as a functional food or dietary supplement. Its potential lies in promoting overall health and well-being by combating oxidative stress and inflammation. However, further research is necessary.

Although this study points to the therapeutic promise of *S. undulata*, there are still significant gaps in terms of clinical utility. Human clinical trials to determine efficacy, formulation optimization (e.g., standardized extracts or bioactive-enriched delivery systems), and mechanistic pathways (e.g., molecular targets for antidiabetic and anti-inflammatory activities) need to be addressed in future studies. Chronic toxicity tests and herb–drug interaction studies must also be performed to ensure safety with prolonged administration. Closing these gaps would combine traditional knowledge with evidence-based practice.

## Figures and Tables

**Table 1:** Ethnomedicinal uses of *S. undulata*.

Species/ Subspecies	Vernacular Name	Geographical Area	Used Parts	Mode of Preparation	Application in Ethnomedicine	Ref
*S. undulata* Vahl	Tamtla/Guiz	Morocco	Flowers	Raw	Treating diabetes mellitus	[22]
*S. undulata* Vahl	Guiz	Morocco	Roots	Fresh	Used against thirst	[23]
*S. undulata* Vahl	Guiz	Tunisia	Tubers/roots	Ashes	Treating burns	[24]
*S. undulata* Vahl	Guiz	Tunisia	-	Decoction	Used as a purgative	[18]
*S. undulata* Vahl	Guiz	Algeria	Leaves Roots	Infusion -	Used as a diuretic, emollient, sudorific, and depurative	[25]
*S. undulata* subsp. *deliciosa* Maire	Guiz	Algeria	-	-	Treating snake bites	[26]
*S. undulata* subsp. *deliciosa* Maire	Elgiz	Libya	Leaves	-	Used as a laxative to treat constipation	[27]
*S. undulata*	Guiz	Tunisia	Leaves and roots	Raw sap (milk) drops in the eye	Against oral inflammation and digestive disorders; antidiarrheal effect and eye treatment	[7]
*S. undulata*	Guiz	Tunisia	Fleshy roots, flowers, and leaves	Fresh	Eaten at the site of collection, as a snack	[28]

**Table 2:** Phytochemical investigations performed on *S. undulata*.

Plant Organ	Extraction Method	Organic Solvent	Yield	Phytochemical Class		Identification Technique	Main Phytochemicals Found	Ref
				Volatile	Phenolic			
Aerial parts	Maceration	Methanol	0.78%		Polyphenols, flavonoids, and tannins	HPLC	Apigenin-7-glucoside, gallic acid, luteolin-7-glucoside and p-coumaric acid	[37]
Roots	Maceration	Methanol	0.86%		Polyphenols, flavonoids, and tannins	HPLC	Luteolin and chlorogenic acid	
Roots	Maceration	Chloroform			Polyphenols	MS, 1H, and 13C NMR, including COSY, HMQC, and HMBC	β-Amyrin acetate, methyl oleanate, stigmasterol, β-sitosterol, galangustin, coumarin-O-β-glycoside, and acteoside	[38]
Aerial parts	Steam distillation	Chloroformic extraction		Oils, hydrocarbons, terpenoids, aromatic compounds, fatty acids, and fatty acid esters		Gas chromatograph, mass spectrometer, and GC/MS	Octadecane, farnesyl acetate, benzyl salicylate, methyl hexadecanoate, heneicosane, methyl octadecanoate, methyl linolenate, tricosane	[11]
Aerial parts	Maceration	Petroleum ether and ethyl acetate	-	Triterpenoids	Flavonoid	Spectroscopic methods (1H-NMR, 13C-NMR, DEPT, HMBC, HSQC, COSY, NOESY), ESI-MS, and EI-MS	Triterpenoids: lupeol, 24-methylenecycloartanol, 3-O-(6-O-acetyl-β-D-glucopyranosyl) β-sitosterol and daucosterol Flavonoid: apigenin	[39]
Roots	Maceration	Chloroform and methanol	-		Polyphenol and flavonoid	Column chromatography on Sephadex LH-20 and MPLC	Acteoside and galangustin	[26]
Roots	Hydrodistillation using a Clevenger-type apparatus	-	-	Essential oil and fatty acids		Gas chromatography–mass spectrometry	Hexadecanoic acid, n-tetradecanoic acid, 9-octadecenoic acid, and 9-hexadecenoic acid	[40]

**Table 3:** Pharmacological and biological properties of *S. undulata*.

Activity Type	Extract Source	Extract Method	Concentration	Positive Control/Reference	Inhibition Percentage (IP)/MIC (µg/mL)	Notes	Ref
Xanthine oxidase	Leaf	Ethanolic (maceration)	50 mg/L	Allopurinol (1 µg/mL)	0 ≤ IP ≤ 38.26	Highest inhibition detected for leaf ethanolic extract due to luteolin-7-O-glucoside.	[18]
Xanthine oxidase	Tubers	Various	50 mg/L	Allopurinol (1 µg/mL)	IP 0	All tuber extracts were inactive, likely due to absence of flavonoids and hydroxycinnamic acids.	
Inflammatory (5-LOX)	Leaf	Ethanolic (ultrasound)	50 mg/L	NDGA	IP 0–14.05	Ethanolic leaf extract showed highest activity; aligns with low anti-inflammatory activity found in *Lantara Camara* essential oil.	
Anti-alpha glucosidase	Flower	Aqueous (ultrasound)	50 mg/L	Acarbose (50 µg/mL)	IP 0–9.77	Showed lower activity; first study, no previous comparisons.	
Anti-alpha amylase	Tuber	Aqueous (maceration)	50 mg/L	Acarbose (50 µg/mL)	IP 0–31.34	Showed some activity; first study, no previous comparisons.	
Anticancer	Aerial parts and tubers	Methanolic (maceration)	4.22 ± 0.06 and 5.89 ± 0.08 mg/mL	-	50%	Extracts from *S. undulata* caused cell death in MCF7 cells through a combination of cell lysis and apoptosis.	[37]
Antibacterial	Aerial part and root	Methanolic extracts	25 mg/mL and 100 mg/mL	Phenol	-	The extract from the above-ground portion of the plant demonstrated antimicrobial effects against three standard bacterial strains: *Pseudomonas aeruginosa*, *Staphylococcus aureus*, and *Escherichia coli*.	[27]
Antifungal	Aerial part and tubers	Methanolic extracts	25 mg/mL and 100 mg/mL	-	-	Both extracts of *S. undulate* did not show any antifungal effect against the fungus *Candida albicans*.	
Laxative	Aerial part	Methanolic extracts	200 and 400 mg/kg	Tween 80	-	The laxative research was conducted solely on the above-ground portion of *S. undulata*. The method involved tracking the transit of a charcoal meal through the gastrointestinal system. The results showed a significant increase, dependent on the dose, in the percentage of the total length of the intestine affected.	
Antibacterial	Aerial parts and tubers	Butanolic, ethyl acetate, petroleum ether, and the product H_2_	-	Gentamicin (10 µg)	MIC 500–2000	The ethyl acetate extract from the aerial part of *S. undulata* exhibited greater activity compared to the petroleum extract. It displayed antibacterial effects against all tested bacterial strains except for *E. coli*. The tuber extract of *S. undulata* exhibited weaker antimicrobial activity when compared to the aerial extracts from the same plant.	[24]
Antifungal	Aerial parts and tubers	Butanolic, ethyl acetate, petroleum ether, and the product H_2_	-	-	IP 0–64.24	The petroleum ether and ethyl acetate extracts of the aerial parts showed potent inhibition against all tested fungi for antifungal activity.	
Antidiabetic	Aerial parts	Aqueous extract	20 mg/kg	Glibenclamide (5 mg/kg)	-	The findings suggest that the hypoglycemic effect of the aqueous extract of *S. undulata*’s aerial parts may be attributed to the improvement of liver structure and function. Furthermore, the dosage used in the study was not found to be toxic.	[33]
Hepatoprotective	Aerial parts	Aqueous extract	20 mg/kg	Glibenclamide (5 mg/kg)	-	Administering the water-based extract from the above-ground portion of *S. undulata* (SUA) orally over a period of 15 days resulted in elevated glycogen contents in the livers of diabetic rats. Additionally, it enhanced the histological architecture of the liver in diabetic rats treated with SUAP. This treatment also had a positive effect on certain biochemical markers, including ALT and ASAT.	
Antidiabetic	Tubers	Aqueous extract	20 mg/kg	Glibenclamide (5 mg/kg)	-	The findings revealed that both single and repeated oral administration of the aqueous extract of *S. undulata* tubers (AERSU) at a dose of 20 mg/kg resulted in significant reductions in the blood glucose levels in both normal and streptozotocin (STZ)-induced diabetic rats. The extract was also found to inhibit the α-amylase activity.	[32]
Hepatoprotective	Tubers	Aqueous extract	20 mg/kg	Glibenclamide (5 mg/kg)	-	Administering AERSU orally over a period of 15 days resulted in an enhancement of the histological architecture of the liver in diabetic rats treated with SUAP. This treatment also had a positive effect on the hepatic biochemical markers ALT and ASAT.	[32]
Antihypertensive	Aerial parts	Aqueous extract	300 mg/kg	Furosemide (5 mg/kg)	-	AESU effectively reduced systolic, diastolic, and mean arterial blood pressure in hypertensive rats. The data analysis showed that AESU exerted its antihypertensive effect through its vasodilatory properties. The vasorelaxation ability of AESU could potentially be mediated by its interaction with receptor-operated calcium channels (ROCCs).	[16]
Hypolipidemic and cardioprotective	Tubers	Aqueous extract	20 mg/kg	Glibenclamide (5 mg/kg)	-	The administration of AERSU led to significant enhancements in the weight of diabetic rats, along with reductions in plasma levels of total cholesterol, triglycerides, and LDL lipoprotein. Moreover, the extract positively influenced the atherogenic index (AI) and coronary risk index (CRI).	[61]

**Table 4:** Antioxidant activity assays performed on different parts of *S. undulata*.

Extract Source	Type of Activity	Extraction Method	Solvent	Concentration	AA (%)/IC_50_ Value/E % (g/L)	Standard Control/Dose	Notes	Ref
Tubers	DPPH scavenging	Ultrasound	Aqueous	50 µg/mL	5.55%	VIT C (4 µg/mL)	Lower antioxidant activity regardless of extraction method or solvent.	[18]
Leaves	DPPH scavenging	Ultrasound	Ethanolic	50 µg/mL	25.06%	VIT C (4 µg/mL)	Highest antioxidant activity; positive correlation with TPC.	
Flowers	DPPH scavenging	Various	Various	50 µg/mL	Varied	VIT C (4 µg/mL)	General pattern: aerial parts and ethanolic extracts showed higher antioxidant activity.	
Tubers	DPPH scavenging	Maceration	Various	50 µg/mL	Lower	VIT C (4 µg/mL)	Lower activity explained by tubers being organ reserve; inulin presence noted.	
Leaves	DPPH scavenging	Maceration	Various	50 µg/mL	Higher	VIT C (4 µg/mL)	Positive correlation with total polyphenolic content (TPC).	
Tubers	DPPH scavenging	Maceration	Chloroform and methanol		0.16 mg	Trolox	Results demonstrated important radical scavenging activity.	[26]
Tubers	CUPRAC	Maceration	Chloroform and methanol		0.23 g/L	Trolox	Results demonstrated important radical scavenging activity.	
Tubers	DPPH scavenging ABTS FRAP	Maceration	Methanol and n-hexane	0.4 mg/mL	DPPH: 0.21 mg/mL FRAP: 0.31 Mm TE/g DW ABTS•+: 1.36 TE/g DW	BHT (0.4 mg/mL) and VIT C (0.4 mg/mL)	The methanolic fraction showed the highest scavenging activity against radicals, exhibiting effective IC_50_ values of 0.14 ± 0.02 mg/mL. Similarly, FRAP and ABTS•+ of methanol extract.	[95]
Tubers and aerial parts	DPPH and FRAP	Maceration	Methanol	0.05−1 mg/mL	Aerial parts DDPH: IC_50_ 0.23 FRAP: OD 0.25 TA (%): 0.61 Tubers DDPH: IC_50_ 0.23 FRAP: IC_50_ 0.11 TA (%): 1.39	Ascorbic acid	The potential to scavenge DPPH and the total antioxidant activity of aerial parts were twofold greater than those of tubers.	[37]
Tubers	DPPH	Decoction	Aqueous	31–500 μg/mL	772.29 μg/mL	BHT 31–500 μg/mL	AERSU possesses potent antioxidant activity.	[61]

CUPRAC: cupric reducing antioxidant capacity, AA: antioxidant activity, TA: total antioxidant, FRAP: ferric reducing antioxidant power, TPC: total polyphenolic content, BHT: butylhydroxytoluene.

## Data Availability

All data are included in the main text.

## Abbreviations

AA, antioxidant activity; AERSU, aqueous extract of roots of *S. undulata*; ALT, alanine transaminase; ASU, aerial parts of *S. undulata*; ASAT, aspartate aminotransferase; BAS, bioavailability score; BBB, blood–brain barrier, BHT, butylhydroxytoluene; CB1 and CB2, cannabinoid receptors 1 and 2; CREB, cAMP response element-binding protein; CUPRAC, cupric reducing antioxidant capacity; FRAP, ferric reducing antioxidant power; GC, gas chromatography; HPLC, high-performance liquid chromatography; IC_50_, half-maximal inhibitory concentration; IL-1β, interleukin-1 beta; LD_50_, lethal dose 50; LPS, lipopolysaccharide; MCF-7, cancer cell line (human breast cancer); MIC, minimum inhibitory concentration; MPLC, medium-pressure liquid chromatography; MS, mass spectrometry; NF-κB, nuclear factor-kappa B; NMR, nuclear magnetic resonance; NOS, Newcastle–Ottawa scale; RACI, relative antioxidant capacity index; RSU, roots of *S. undulata*; STZ, streptozotocin; SUAP, aerial parts of *S. undulata*; TA, total antioxidant; TNF-alpha, tumor necrosis factor alpha; TPC, total polyphenolic content; UV, ultraviolet; VEGFR-2, vascular endothelial growth factor receptor 2.
